# GENDER DIFFERENCES IN INTERACTIONS AND WELL-BEING AMONG HOSPITALIZED PATIENTS LIVING WITH DEMENTIA

**DOI:** 10.1093/geroni/igac059.382

**Published:** 2022-12-20

**Authors:** Anju Paudel, Marie Boltz, Barbara Resnick

**Affiliations:** Penn State Ross and Carol Nese College of Nursing, State College, Pennsylvania, United States; Penn State, Pennsylvania State University, Pennsylvania, United States; University of Maryland, Baltimore, Maryland, United States

## Abstract

While the incidence of dementia is generally higher in women compared to men, gender differences in interactions and well-being in dementia is still unclear. This study examined gender differences in interactions and well-being among hospitalized patients living with dementia. A total of 140 hospitalized patients (53% female and 47% male) were included in the analysis. On average, the participants were 81.43 years old (SD= 8.29), had positive interactions with staff based on higher scores on Quality of Interaction Schedule, QUIS (5.81, SD= 1.36), and fair emotional well-being based on lower scores on Cornell Scale for Depression in Dementia, CSDD (7.79, SD= 5.59). Although men seemed to have more positive interactions (male=6.07, SD=1.13; female=5.59, SD=1.51) and greater wellbeing (male=7.52, SD=4.77; female=8.03, SD=6.25) than women, there were no statistically significant gender differences observed in linear models with appropriate covariates. Future work should continue to explore gender differences in interactions and well-being.

